# 2-Diethyl­amino-6-methyl­pyrimidin-4(3*H*)-one

**DOI:** 10.1107/S1600536811040451

**Published:** 2011-10-08

**Authors:** Mei-Yi Wang

**Affiliations:** a College of Chemistry and Chemical Engineering, The North University for Ethnics, Yinchuan 750021, People’s Republic of China

## Abstract

The title compound, C_9_H_15_N_3_O, contains four mol­ecules (*A*, *B*, *C* and *D*) in the asymmetric unit. In the crystal, the *A*+*A* and *D*+*D* pairs form inversion dimers linked by pairs of N—H⋯O hydrogen bonds. The *B*+*C* pairing is linked by the same bonds. The dimers are further linked by weak C—H⋯O inter­actions.

## Related literature

For further details of the synthesis, see: Huang *et al.* (2007 ▶). For the biological activity of related compounds, see, for example: Atul *et al.* (2010 ▶); Liu, Jian & Tan (2011 ▶); Liu, Jian, Tan *et al.* (2011 ▶).
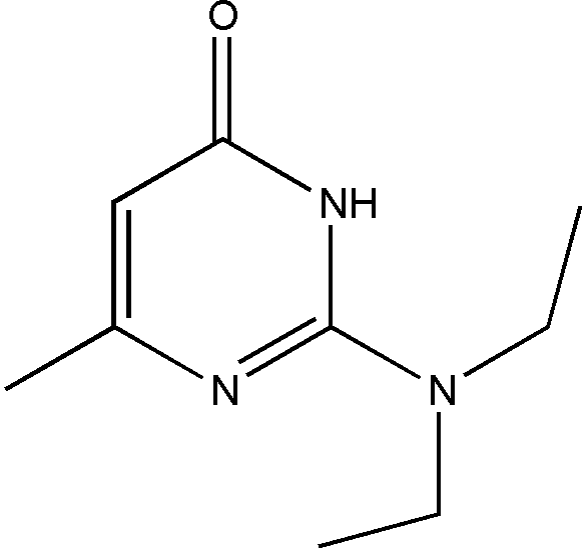


## Experimental

### 

#### Crystal data

C_9_H_15_N_3_O
*M* *_r_* = 181.24
Triclinic, 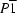

*a* = 11.799 (5) Å
*b* = 12.136 (5) Å
*c* = 15.023 (5) Å
α = 92.753 (5)°
β = 94.538 (6)°
γ = 112.103 (5)°
*V* = 1979.6 (13) Å^3^
*Z* = 8
Mo *K*α radiation
μ = 0.08 mm^−1^
*T* = 113 K
0.20 × 0.18 × 0.10 mm

#### Data collection

Rigaku Saturn CCD area-detector diffractometer
Absorption correction: multi-scan (*CrystalClear*; Rigaku/MSC, 2005 ▶) *T*_min_ = 0.984, *T*_max_ = 0.992
20802 measured reflections
9288 independent reflections
6322 reflections with *I* > 2σ(*I*)
*R*_int_ = 0.033

#### Refinement

*R*[*F* ^2^ > 2σ(*F* ^2^)] = 0.045
*wR*(*F* ^2^) = 0.097
*S* = 0.96
9288 reflections
481 parameters
H-atom parameters constrained
Δρ_max_ = 0.19 e Å^−3^
Δρ_min_ = −0.20 e Å^−3^

### 

Data collection: *CrystalClear* (Rigaku/MSC, 2005 ▶); cell refinement: *CrystalClear*; data reduction: *CrystalClear*; program(s) used to solve structure: *SHELXS97* (Sheldrick, 2008 ▶); program(s) used to refine structure: *SHELXL97* (Sheldrick, 2008 ▶); molecular graphics: *SHELXTL* (Sheldrick, 2008 ▶); software used to prepare material for publication: *CrystalStructure* (Rigaku/MSC, 2005 ▶).

## Supplementary Material

Crystal structure: contains datablock(s) global, I. DOI: 10.1107/S1600536811040451/hb6420sup1.cif

Structure factors: contains datablock(s) I. DOI: 10.1107/S1600536811040451/hb6420Isup2.hkl

Supplementary material file. DOI: 10.1107/S1600536811040451/hb6420Isup3.cml

Additional supplementary materials:  crystallographic information; 3D view; checkCIF report

## Figures and Tables

**Table 1:** Hydrogen-bond geometry (Å, °)

*D*—H⋯*A*	*D*—H	H⋯*A*	*D*⋯*A*	*D*—H⋯*A*
N1—H1*A*⋯O1^i^	0.88	2.02	2.8420 (19)	156
N4—H4*A*⋯O3	0.88	1.98	2.8407 (18)	164
N8—H8*C*⋯O2	0.88	1.99	2.8420 (18)	162
N10—H10*A*⋯O4^ii^	0.88	1.98	2.8248 (18)	162
C6—H6*A*⋯O1^i^	0.99	2.22	3.116 (2)	150
C15—H15*A*⋯O3	0.99	2.35	3.120 (2)	134
C16—H16*B*⋯O1	0.98	2.58	3.507 (2)	158
C26—H26*A*⋯O2	0.99	2.31	3.130 (2)	139
C33—H33*A*⋯O4^ii^	0.99	2.42	3.143 (2)	130

Symmetry codes: (i) 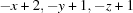; (ii) 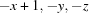.

**Fig. 1.**
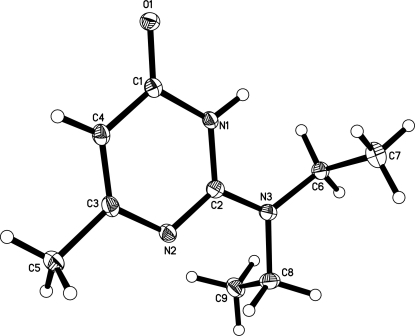
The structure of one of the moleucles of (I) showing 30% displacement ellipsoids.

**Fig. 2.**
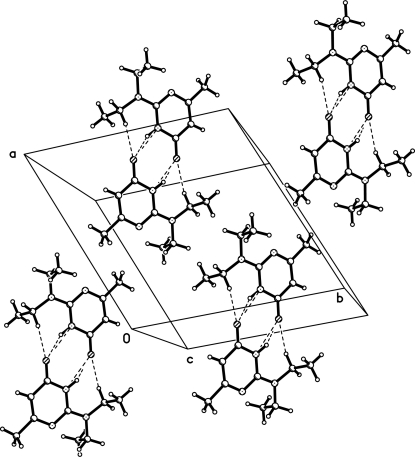
The crystal packing for (I).

## supplementary crystallographic information

### Experimental

1,1-Diethylguanidine and ethyl 3-oxobutanoate was stirred in EtOH/NaOH solution. The mixture was refluxed for 1 h. The product was collected. Colourless prisms were obtained from EtOH soltion.

### Refinement

All the H atoms were positioned geometrically (C—H = 0.93–0.97 Å) and refined as riding with *U*_iso_(H) = 1.2*U*_eq_(C) or 1.5*U*_eq_(methyl C).

### Crystal data

**Table:**

C_9_H_15_N_3_O	*Z* = 8
*M**_r_* = 181.24	*F*(000) = 784
Triclinic, *P*1	*D*_x_ = 1.216 Mg m^−^^3^
*a* = 11.799 (5) Å	Mo *K*α radiation, λ = 0.71073 Å
*b* = 12.136 (5) Å	Cell parameters from 6611 reflections
*c* = 15.023 (5) Å	θ = 1.8–27.9°
α = 92.753 (5)°	µ = 0.08 mm^−^^1^
β = 94.538 (6)°	*T* = 113 K
γ = 112.103 (5)°	Prism, colorless
*V* = 1979.6 (13) Å^3^	0.20 × 0.18 × 0.10 mm

### Data collection

**Table:**

Rigaku Saturn CCD area-detector diffractometer	9288 independent reflections
Radiation source: rotating anode	6322 reflections with *I* > 2σ(*I*)
multilayer	*R*_int_ = 0.033
Detector resolution: 14.63 pixels mm^-1^	θ_max_ = 27.9°, θ_min_ = 1.8°
ω and φ scans	*h* = −15→15
Absorption correction: multi-scan (*CrystalClear*; Rigaku/MSC, 2005)	*k* = −15→15
*T*_min_ = 0.984, *T*_max_ = 0.992	*l* = −14→19
20802 measured reflections	

### Refinement

**Table:**

Refinement on *F*^2^	Primary atom site location: structure-invariant direct methods
Least-squares matrix: full	Secondary atom site location: difference Fourier map
*R*[*F*^2^ > 2σ(*F*^2^)] = 0.045	Hydrogen site location: inferred from neighbouring sites
*wR*(*F*^2^) = 0.097	H-atom parameters constrained
*S* = 0.96	*w* = 1/[σ^2^(*F*_o_^2^) + (0.0355*P*)^2^] where *P* = (*F*_o_^2^ + 2*F*_c_^2^)/3
9288 reflections	(Δ/σ)_max_ = 0.001
481 parameters	Δρ_max_ = 0.19 e Å^−^^3^
0 restraints	Δρ_min_ = −0.20 e Å^−^^3^

### Special details

**Table:**

Geometry. All e.s.d.'s (except the e.s.d. in the dihedral angle between two l.s. planes) are estimated using the full covariance matrix. The cell e.s.d.'s are taken into account individually in the estimation of e.s.d.'s in distances, angles and torsion angles; correlations between e.s.d.'s in cell parameters are only used when they are defined by crystal symmetry. An approximate (isotropic) treatment of cell e.s.d.'s is used for estimating e.s.d.'s involving l.s. planes.
Refinement. Refinement of *F*^2^ against ALL reflections. The weighted *R*-factor *wR* and goodness of fit *S* are based on *F*^2^, conventional *R*-factors *R* are based on *F*, with *F* set to zero for negative *F*^2^. The threshold expression of *F*^2^ > σ(*F*^2^) is used only for calculating *R*-factors(gt) *etc*. and is not relevant to the choice of reflections for refinement. *R*-factors based on *F*^2^ are statistically about twice as large as those based on *F*, and *R*- factors based on ALL data will be even larger.

### Fractional atomic coordinates and isotropic or equivalent isotropic displacement parameters (Å^2^)

**Table:**

	*x*	*y*	*z*	*U*_iso_*/*U*_eq_	
O1	0.99183 (8)	0.40717 (8)	0.41209 (6)	0.0251 (2)	
O2	0.72491 (9)	0.84710 (8)	0.18851 (6)	0.0301 (2)	
O3	0.75913 (8)	0.63637 (8)	0.30675 (6)	0.0274 (2)	
O4	0.49910 (8)	0.07510 (8)	0.09424 (6)	0.0274 (2)	
N1	0.82458 (10)	0.39755 (9)	0.48181 (7)	0.0194 (2)	
H1A	0.8668	0.4686	0.5096	0.023*	
N2	0.63604 (10)	0.23127 (9)	0.45981 (7)	0.0232 (3)	
N3	0.65972 (10)	0.38934 (9)	0.56118 (7)	0.0231 (3)	
N4	0.89914 (10)	0.87803 (9)	0.27973 (7)	0.0203 (3)	
H4A	0.8694	0.8027	0.2916	0.024*	
N5	1.06587 (10)	1.06548 (9)	0.30369 (7)	0.0203 (3)	
N6	1.07319 (10)	0.90590 (9)	0.37854 (7)	0.0224 (3)	
N7	0.41150 (10)	0.42370 (9)	0.20112 (7)	0.0215 (3)	
N8	0.58723 (9)	0.60348 (9)	0.21177 (7)	0.0205 (3)	
H8C	0.6222	0.6746	0.1925	0.025*	
N9	0.41080 (10)	0.57771 (9)	0.11655 (7)	0.0223 (3)	
N10	0.67058 (9)	0.11616 (9)	0.02141 (7)	0.0191 (2)	
H10A	0.6319	0.0523	−0.0159	0.023*	
N11	0.85329 (10)	0.28537 (9)	0.06299 (7)	0.0208 (3)	
N12	0.84578 (10)	0.15135 (9)	−0.05414 (7)	0.0238 (3)	
C1	0.88085 (12)	0.35068 (11)	0.42104 (8)	0.0206 (3)	
C2	0.70603 (12)	0.33780 (11)	0.50029 (9)	0.0207 (3)	
C3	0.68696 (12)	0.18461 (11)	0.39732 (9)	0.0233 (3)	
C4	0.80429 (12)	0.23978 (11)	0.37574 (9)	0.0230 (3)	
H4	0.8342	0.2037	0.3305	0.028*	
C5	0.60347 (13)	0.06516 (12)	0.35259 (10)	0.0342 (4)	
H5A	0.5395	0.0749	0.3117	0.051*	
H5B	0.5650	0.0121	0.3982	0.051*	
H5C	0.6513	0.0304	0.3187	0.051*	
C6	0.72752 (12)	0.50937 (11)	0.60599 (9)	0.0239 (3)	
H6A	0.8147	0.5207	0.6206	0.029*	
H6B	0.6929	0.5165	0.6629	0.029*	
C7	0.72098 (14)	0.60672 (12)	0.54788 (10)	0.0361 (4)	
H7A	0.7538	0.5991	0.4910	0.054*	
H7B	0.7698	0.6852	0.5792	0.054*	
H7C	0.6352	0.5986	0.5361	0.054*	
C8	0.53807 (12)	0.32129 (12)	0.58906 (9)	0.0287 (3)	
H8A	0.4851	0.2675	0.5377	0.034*	
H8B	0.4992	0.3770	0.6075	0.034*	
C9	0.54776 (14)	0.24807 (12)	0.66640 (9)	0.0342 (4)	
H9A	0.5819	0.1898	0.6471	0.051*	
H9B	0.4658	0.2061	0.6852	0.051*	
H9C	0.6018	0.3011	0.7167	0.051*	
C10	0.82874 (12)	0.91843 (12)	0.22073 (9)	0.0217 (3)	
C11	1.01310 (12)	0.95142 (11)	0.32001 (8)	0.0194 (3)	
C12	1.00049 (12)	1.10774 (11)	0.24514 (8)	0.0206 (3)	
C13	0.88573 (12)	1.03962 (11)	0.20382 (9)	0.0224 (3)	
H13	0.8444	1.0739	0.1638	0.027*	
C14	1.06188 (12)	1.23722 (11)	0.23059 (9)	0.0251 (3)	
H14A	1.0148	1.2570	0.1816	0.038*	
H14B	1.1455	1.2535	0.2151	0.038*	
H14C	1.0655	1.2859	0.2855	0.038*	
C15	1.01969 (12)	0.78372 (11)	0.40542 (9)	0.0240 (3)	
H15A	0.9296	0.7599	0.4048	0.029*	
H15B	1.0534	0.7821	0.4676	0.029*	
C16	1.04530 (13)	0.69369 (11)	0.34475 (9)	0.0294 (3)	
H16A	1.0100	0.6930	0.2834	0.044*	
H16B	1.0081	0.6141	0.3660	0.044*	
H16C	1.1344	0.7160	0.3458	0.044*	
C17	1.19429 (12)	0.98323 (12)	0.42367 (9)	0.0249 (3)	
H17A	1.2378	1.0437	0.3832	0.030*	
H17B	1.2438	0.9345	0.4364	0.030*	
C18	1.18373 (13)	1.04601 (12)	0.51076 (9)	0.0338 (4)	
H18A	1.1385	1.0976	0.4980	0.051*	
H18B	1.2662	1.0945	0.5398	0.051*	
H18C	1.1396	0.9864	0.5507	0.051*	
C19	0.65370 (12)	0.56663 (11)	0.27664 (9)	0.0207 (3)	
C20	0.46966 (12)	0.53325 (11)	0.17699 (8)	0.0191 (3)	
C21	0.47506 (12)	0.38272 (11)	0.26201 (8)	0.0206 (3)	
C22	0.59122 (12)	0.44956 (11)	0.30103 (9)	0.0219 (3)	
H22	0.6300	0.4173	0.3446	0.026*	
C23	0.40973 (12)	0.25534 (11)	0.28142 (9)	0.0272 (3)	
H23A	0.3253	0.2422	0.2933	0.041*	
H23B	0.4532	0.2384	0.3340	0.041*	
H23C	0.4080	0.2023	0.2296	0.041*	
C24	0.28663 (12)	0.50323 (12)	0.07508 (9)	0.0267 (3)	
H24A	0.2734	0.4181	0.0787	0.032*	
H24B	0.2798	0.5176	0.0109	0.032*	
C25	0.18751 (12)	0.52887 (13)	0.11996 (10)	0.0337 (4)	
H25A	0.1905	0.5100	0.1826	0.051*	
H25B	0.1067	0.4798	0.0886	0.051*	
H25C	0.2013	0.6135	0.1177	0.051*	
C26	0.46937 (12)	0.69518 (11)	0.08266 (9)	0.0241 (3)	
H26A	0.5203	0.7531	0.1327	0.029*	
H26B	0.4049	0.7229	0.0590	0.029*	
C27	0.54991 (13)	0.69227 (13)	0.00906 (9)	0.0344 (4)	
H27A	0.6145	0.6658	0.0324	0.052*	
H27B	0.5877	0.7724	−0.0112	0.052*	
H27C	0.4994	0.6368	−0.0414	0.052*	
C28	0.60785 (12)	0.14249 (11)	0.08855 (9)	0.0215 (3)	
C29	0.79042 (12)	0.18576 (11)	0.01091 (8)	0.0195 (3)	
C30	0.79382 (12)	0.31562 (11)	0.12831 (9)	0.0211 (3)	
C31	0.67636 (12)	0.24850 (11)	0.14345 (9)	0.0226 (3)	
H31	0.6403	0.2730	0.1910	0.027*	
C32	0.86739 (12)	0.43106 (11)	0.18306 (9)	0.0274 (3)	
H32A	0.9365	0.4228	0.2188	0.041*	
H32B	0.8148	0.4509	0.2230	0.041*	
H32C	0.8989	0.4948	0.1431	0.041*	
C33	0.78135 (12)	0.04901 (12)	−0.12002 (9)	0.0267 (3)	
H33A	0.7212	−0.0157	−0.0905	0.032*	
H33B	0.8415	0.0185	−0.1426	0.032*	
C34	0.71434 (13)	0.08240 (12)	−0.19856 (9)	0.0333 (4)	
H34A	0.6490	0.1049	−0.1772	0.050*	
H34B	0.6782	0.0139	−0.2434	0.050*	
H34C	0.7727	0.1498	−0.2256	0.050*	
C35	0.97244 (12)	0.22543 (13)	−0.06902 (10)	0.0315 (4)	
H35A	0.9925	0.3080	−0.0432	0.038*	
H35B	0.9789	0.2279	−0.1343	0.038*	
C36	1.06510 (14)	0.17855 (14)	−0.02713 (10)	0.0439 (4)	
H36A	1.0639	0.1822	0.0381	0.066*	
H36B	1.1476	0.2276	−0.0418	0.066*	
H36C	1.0436	0.0957	−0.0506	0.066*	

### Atomic displacement parameters (Å^2^)

**Table:**

	*U*^11^	*U*^22^	*U*^33^	*U*^12^	*U*^13^	*U*^23^
O1	0.0219 (5)	0.0236 (5)	0.0266 (5)	0.0051 (4)	0.0046 (4)	−0.0022 (4)
O2	0.0239 (5)	0.0237 (5)	0.0364 (6)	0.0037 (4)	−0.0077 (4)	0.0048 (4)
O3	0.0207 (5)	0.0229 (5)	0.0346 (6)	0.0048 (4)	−0.0045 (4)	0.0046 (4)
O4	0.0199 (5)	0.0280 (5)	0.0287 (6)	0.0026 (4)	0.0066 (4)	−0.0022 (4)
N1	0.0208 (6)	0.0154 (6)	0.0205 (6)	0.0056 (5)	0.0012 (5)	−0.0011 (5)
N2	0.0214 (6)	0.0188 (6)	0.0273 (7)	0.0067 (5)	−0.0026 (5)	−0.0023 (5)
N3	0.0200 (6)	0.0210 (6)	0.0264 (7)	0.0054 (5)	0.0043 (5)	0.0000 (5)
N4	0.0214 (6)	0.0155 (6)	0.0226 (6)	0.0057 (5)	−0.0006 (5)	0.0035 (5)
N5	0.0213 (6)	0.0173 (6)	0.0219 (6)	0.0070 (5)	0.0011 (5)	0.0016 (5)
N6	0.0211 (6)	0.0190 (6)	0.0253 (6)	0.0060 (5)	−0.0019 (5)	0.0050 (5)
N7	0.0199 (6)	0.0209 (6)	0.0237 (6)	0.0075 (5)	0.0029 (5)	0.0034 (5)
N8	0.0187 (6)	0.0189 (6)	0.0238 (6)	0.0067 (5)	0.0016 (5)	0.0052 (5)
N9	0.0174 (6)	0.0224 (6)	0.0254 (6)	0.0061 (5)	−0.0008 (5)	0.0049 (5)
N10	0.0176 (6)	0.0183 (6)	0.0191 (6)	0.0044 (5)	0.0017 (5)	−0.0006 (5)
N11	0.0186 (6)	0.0209 (6)	0.0215 (6)	0.0065 (5)	0.0008 (5)	0.0001 (5)
N12	0.0195 (6)	0.0243 (6)	0.0243 (6)	0.0045 (5)	0.0055 (5)	−0.0033 (5)
C1	0.0236 (7)	0.0197 (7)	0.0189 (7)	0.0090 (6)	0.0002 (6)	0.0029 (6)
C2	0.0201 (7)	0.0213 (7)	0.0209 (7)	0.0086 (6)	−0.0016 (6)	0.0044 (6)
C3	0.0253 (8)	0.0180 (7)	0.0260 (8)	0.0091 (6)	−0.0048 (6)	−0.0005 (6)
C4	0.0265 (8)	0.0207 (7)	0.0212 (7)	0.0095 (6)	−0.0004 (6)	−0.0030 (6)
C5	0.0257 (8)	0.0239 (8)	0.0476 (10)	0.0062 (6)	−0.0046 (7)	−0.0084 (7)
C6	0.0237 (7)	0.0227 (7)	0.0244 (8)	0.0080 (6)	0.0028 (6)	−0.0016 (6)
C7	0.0460 (10)	0.0270 (8)	0.0350 (9)	0.0142 (7)	0.0019 (7)	0.0025 (7)
C8	0.0201 (7)	0.0304 (8)	0.0322 (9)	0.0059 (6)	0.0044 (6)	−0.0023 (7)
C9	0.0361 (9)	0.0243 (8)	0.0398 (9)	0.0069 (7)	0.0137 (7)	0.0017 (7)
C10	0.0214 (7)	0.0226 (7)	0.0213 (7)	0.0092 (6)	0.0000 (6)	0.0011 (6)
C11	0.0191 (7)	0.0211 (7)	0.0181 (7)	0.0079 (6)	0.0024 (5)	0.0000 (6)
C12	0.0243 (7)	0.0209 (7)	0.0183 (7)	0.0104 (6)	0.0031 (6)	0.0017 (6)
C13	0.0248 (7)	0.0201 (7)	0.0226 (8)	0.0093 (6)	−0.0009 (6)	0.0023 (6)
C14	0.0247 (8)	0.0200 (7)	0.0289 (8)	0.0071 (6)	−0.0007 (6)	0.0033 (6)
C15	0.0250 (8)	0.0217 (7)	0.0228 (8)	0.0066 (6)	−0.0034 (6)	0.0071 (6)
C16	0.0305 (8)	0.0223 (8)	0.0344 (9)	0.0094 (6)	−0.0014 (7)	0.0064 (6)
C17	0.0202 (7)	0.0247 (8)	0.0277 (8)	0.0070 (6)	−0.0024 (6)	0.0047 (6)
C18	0.0319 (9)	0.0316 (9)	0.0329 (9)	0.0089 (7)	−0.0052 (7)	−0.0025 (7)
C19	0.0194 (7)	0.0214 (7)	0.0223 (7)	0.0091 (6)	0.0017 (6)	0.0014 (6)
C20	0.0184 (7)	0.0211 (7)	0.0192 (7)	0.0089 (6)	0.0030 (5)	0.0003 (6)
C21	0.0215 (7)	0.0208 (7)	0.0212 (7)	0.0097 (6)	0.0047 (6)	0.0009 (6)
C22	0.0218 (7)	0.0216 (7)	0.0230 (7)	0.0092 (6)	0.0000 (6)	0.0031 (6)
C23	0.0231 (8)	0.0238 (8)	0.0345 (8)	0.0082 (6)	0.0028 (6)	0.0055 (6)
C24	0.0206 (7)	0.0283 (8)	0.0285 (8)	0.0074 (6)	−0.0045 (6)	0.0027 (6)
C25	0.0204 (8)	0.0369 (9)	0.0418 (9)	0.0086 (7)	0.0013 (7)	0.0070 (7)
C26	0.0217 (7)	0.0256 (8)	0.0262 (8)	0.0102 (6)	0.0012 (6)	0.0065 (6)
C27	0.0326 (9)	0.0402 (9)	0.0334 (9)	0.0156 (7)	0.0079 (7)	0.0111 (7)
C28	0.0204 (7)	0.0231 (7)	0.0215 (7)	0.0086 (6)	0.0028 (6)	0.0028 (6)
C29	0.0176 (7)	0.0210 (7)	0.0205 (7)	0.0075 (6)	0.0019 (5)	0.0057 (6)
C30	0.0216 (7)	0.0222 (7)	0.0207 (7)	0.0104 (6)	−0.0006 (6)	0.0020 (6)
C31	0.0213 (7)	0.0250 (8)	0.0210 (7)	0.0084 (6)	0.0037 (6)	−0.0025 (6)
C32	0.0233 (8)	0.0241 (8)	0.0324 (8)	0.0072 (6)	0.0020 (6)	−0.0036 (6)
C33	0.0241 (8)	0.0257 (8)	0.0275 (8)	0.0065 (6)	0.0065 (6)	−0.0043 (6)
C34	0.0325 (9)	0.0325 (9)	0.0266 (8)	0.0026 (7)	0.0049 (7)	0.0007 (7)
C35	0.0217 (8)	0.0321 (9)	0.0328 (9)	0.0007 (6)	0.0102 (6)	−0.0031 (7)
C36	0.0233 (8)	0.0598 (12)	0.0443 (10)	0.0130 (8)	0.0035 (7)	−0.0119 (9)

### Geometric parameters (Å, °)

**Table:**

O1—C1	1.2506 (15)	C12—C13	1.3702 (18)
O2—C10	1.2459 (15)	C12—C14	1.4977 (17)
O3—C19	1.2451 (15)	C13—H13	0.9500
O4—C28	1.2477 (15)	C14—H14A	0.9800
N1—C2	1.3699 (16)	C14—H14B	0.9800
N1—C1	1.3886 (17)	C14—H14C	0.9800
N1—H1A	0.8800	C15—C16	1.5206 (19)
N2—C2	1.3284 (16)	C15—H15A	0.9900
N2—C3	1.3628 (17)	C15—H15B	0.9900
N3—C2	1.3445 (17)	C16—H16A	0.9800
N3—C8	1.4677 (16)	C16—H16B	0.9800
N3—C6	1.4692 (16)	C16—H16C	0.9800
N4—C11	1.3732 (16)	C17—C18	1.5196 (19)
N4—C10	1.3956 (16)	C17—H17A	0.9900
N4—H4A	0.8800	C17—H17B	0.9900
N5—C11	1.3311 (16)	C18—H18A	0.9800
N5—C12	1.3658 (16)	C18—H18B	0.9800
N6—C11	1.3504 (16)	C18—H18C	0.9800
N6—C15	1.4676 (16)	C19—C22	1.4145 (17)
N6—C17	1.4691 (16)	C21—C22	1.3677 (17)
N7—C20	1.3301 (16)	C21—C23	1.5004 (17)
N7—C21	1.3645 (16)	C22—H22	0.9500
N8—C20	1.3698 (16)	C23—H23A	0.9800
N8—C19	1.3963 (16)	C23—H23B	0.9800
N8—H8C	0.8800	C23—H23C	0.9800
N9—C20	1.3517 (16)	C24—C25	1.5158 (19)
N9—C26	1.4673 (16)	C24—H24A	0.9900
N9—C24	1.4685 (16)	C24—H24B	0.9900
N10—C29	1.3735 (16)	C25—H25A	0.9800
N10—C28	1.3902 (16)	C25—H25B	0.9800
N10—H10A	0.8800	C25—H25C	0.9800
N11—C29	1.3291 (15)	C26—C27	1.5213 (19)
N11—C30	1.3632 (17)	C26—H26A	0.9900
N12—C29	1.3501 (17)	C26—H26B	0.9900
N12—C33	1.4671 (16)	C27—H27A	0.9800
N12—C35	1.4672 (16)	C27—H27B	0.9800
C1—C4	1.4107 (17)	C27—H27C	0.9800
C3—C4	1.3653 (18)	C28—C31	1.4138 (17)
C3—C5	1.5002 (18)	C30—C31	1.3615 (18)
C4—H4	0.9500	C30—C32	1.5013 (17)
C5—H5A	0.9800	C31—H31	0.9500
C5—H5B	0.9800	C32—H32A	0.9800
C5—H5C	0.9800	C32—H32B	0.9800
C6—C7	1.5234 (19)	C32—H32C	0.9800
C6—H6A	0.9900	C33—C34	1.5234 (19)
C6—H6B	0.9900	C33—H33A	0.9900
C7—H7A	0.9800	C33—H33B	0.9900
C7—H7B	0.9800	C34—H34A	0.9800
C7—H7C	0.9800	C34—H34B	0.9800
C8—C9	1.5190 (19)	C34—H34C	0.9800
C8—H8A	0.9900	C35—C36	1.519 (2)
C8—H8B	0.9900	C35—H35A	0.9900
C9—H9A	0.9800	C35—H35B	0.9900
C9—H9B	0.9800	C36—H36A	0.9800
C9—H9C	0.9800	C36—H36B	0.9800
C10—C13	1.4136 (18)	C36—H36C	0.9800
			
C2—N1—C1	122.70 (11)	H16A—C16—H16C	109.5
C2—N1—H1A	118.7	H16B—C16—H16C	109.5
C1—N1—H1A	118.7	N6—C17—C18	111.92 (12)
C2—N2—C3	116.78 (12)	N6—C17—H17A	109.2
C2—N3—C8	119.26 (11)	C18—C17—H17A	109.2
C2—N3—C6	123.42 (11)	N6—C17—H17B	109.2
C8—N3—C6	117.23 (11)	C18—C17—H17B	109.2
C11—N4—C10	122.46 (11)	H17A—C17—H17B	107.9
C11—N4—H4A	118.8	C17—C18—H18A	109.5
C10—N4—H4A	118.8	C17—C18—H18B	109.5
C11—N5—C12	116.80 (11)	H18A—C18—H18B	109.5
C11—N6—C15	123.21 (11)	C17—C18—H18C	109.5
C11—N6—C17	119.55 (11)	H18A—C18—H18C	109.5
C15—N6—C17	117.07 (10)	H18B—C18—H18C	109.5
C20—N7—C21	116.87 (11)	O3—C19—N8	119.03 (12)
C20—N8—C19	122.58 (11)	O3—C19—C22	126.50 (12)
C20—N8—H8C	118.7	N8—C19—C22	114.47 (11)
C19—N8—H8C	118.7	N7—C20—N9	119.12 (12)
C20—N9—C26	123.02 (11)	N7—C20—N8	122.33 (12)
C20—N9—C24	120.05 (11)	N9—C20—N8	118.55 (12)
C26—N9—C24	116.67 (11)	N7—C21—C22	123.59 (12)
C29—N10—C28	122.68 (11)	N7—C21—C23	114.92 (11)
C29—N10—H10A	118.7	C22—C21—C23	121.45 (12)
C28—N10—H10A	118.7	C21—C22—C19	120.09 (12)
C29—N11—C30	117.02 (11)	C21—C22—H22	120.0
C29—N12—C33	123.36 (11)	C19—C22—H22	120.0
C29—N12—C35	120.25 (11)	C21—C23—H23A	109.5
C33—N12—C35	115.98 (11)	C21—C23—H23B	109.5
O1—C1—N1	118.90 (11)	H23A—C23—H23B	109.5
O1—C1—C4	126.25 (13)	C21—C23—H23C	109.5
N1—C1—C4	114.84 (12)	H23A—C23—H23C	109.5
N2—C2—N3	119.39 (12)	H23B—C23—H23C	109.5
N2—C2—N1	122.02 (13)	N9—C24—C25	112.31 (11)
N3—C2—N1	118.59 (11)	N9—C24—H24A	109.1
N2—C3—C4	123.99 (12)	C25—C24—H24A	109.1
N2—C3—C5	114.65 (13)	N9—C24—H24B	109.1
C4—C3—C5	121.36 (13)	C25—C24—H24B	109.1
C3—C4—C1	119.56 (13)	H24A—C24—H24B	107.9
C3—C4—H4	120.2	C24—C25—H25A	109.5
C1—C4—H4	120.2	C24—C25—H25B	109.5
C3—C5—H5A	109.5	H25A—C25—H25B	109.5
C3—C5—H5B	109.5	C24—C25—H25C	109.5
H5A—C5—H5B	109.5	H25A—C25—H25C	109.5
C3—C5—H5C	109.5	H25B—C25—H25C	109.5
H5A—C5—H5C	109.5	N9—C26—C27	112.16 (11)
H5B—C5—H5C	109.5	N9—C26—H26A	109.2
N3—C6—C7	112.06 (11)	C27—C26—H26A	109.2
N3—C6—H6A	109.2	N9—C26—H26B	109.2
C7—C6—H6A	109.2	C27—C26—H26B	109.2
N3—C6—H6B	109.2	H26A—C26—H26B	107.9
C7—C6—H6B	109.2	C26—C27—H27A	109.5
H6A—C6—H6B	107.9	C26—C27—H27B	109.5
C6—C7—H7A	109.5	H27A—C27—H27B	109.5
C6—C7—H7B	109.5	C26—C27—H27C	109.5
H7A—C7—H7B	109.5	H27A—C27—H27C	109.5
C6—C7—H7C	109.5	H27B—C27—H27C	109.5
H7A—C7—H7C	109.5	O4—C28—N10	119.22 (12)
H7B—C7—H7C	109.5	O4—C28—C31	126.28 (13)
N3—C8—C9	111.14 (12)	N10—C28—C31	114.48 (12)
N3—C8—H8A	109.4	N11—C29—N12	119.22 (12)
C9—C8—H8A	109.4	N11—C29—N10	121.97 (12)
N3—C8—H8B	109.4	N12—C29—N10	118.80 (11)
C9—C8—H8B	109.4	C31—C30—N11	123.61 (12)
H8A—C8—H8B	108.0	C31—C30—C32	121.54 (12)
C8—C9—H9A	109.5	N11—C30—C32	114.85 (12)
C8—C9—H9B	109.5	C30—C31—C28	120.19 (13)
H9A—C9—H9B	109.5	C30—C31—H31	119.9
C8—C9—H9C	109.5	C28—C31—H31	119.9
H9A—C9—H9C	109.5	C30—C32—H32A	109.5
H9B—C9—H9C	109.5	C30—C32—H32B	109.5
O2—C10—N4	118.68 (12)	H32A—C32—H32B	109.5
O2—C10—C13	126.68 (13)	C30—C32—H32C	109.5
N4—C10—C13	114.64 (12)	H32A—C32—H32C	109.5
N5—C11—N6	119.06 (12)	H32B—C32—H32C	109.5
N5—C11—N4	122.39 (12)	N12—C33—C34	111.74 (12)
N6—C11—N4	118.55 (12)	N12—C33—H33A	109.3
N5—C12—C13	123.62 (12)	C34—C33—H33A	109.3
N5—C12—C14	115.00 (11)	N12—C33—H33B	109.3
C13—C12—C14	121.36 (12)	C34—C33—H33B	109.3
C12—C13—C10	120.07 (12)	H33A—C33—H33B	107.9
C12—C13—H13	120.0	C33—C34—H34A	109.5
C10—C13—H13	120.0	C33—C34—H34B	109.5
C12—C14—H14A	109.5	H34A—C34—H34B	109.5
C12—C14—H14B	109.5	C33—C34—H34C	109.5
H14A—C14—H14B	109.5	H34A—C34—H34C	109.5
C12—C14—H14C	109.5	H34B—C34—H34C	109.5
H14A—C14—H14C	109.5	N12—C35—C36	112.23 (13)
H14B—C14—H14C	109.5	N12—C35—H35A	109.2
N6—C15—C16	112.98 (12)	C36—C35—H35A	109.2
N6—C15—H15A	109.0	N12—C35—H35B	109.2
C16—C15—H15A	109.0	C36—C35—H35B	109.2
N6—C15—H15B	109.0	H35A—C35—H35B	107.9
C16—C15—H15B	109.0	C35—C36—H36A	109.5
H15A—C15—H15B	107.8	C35—C36—H36B	109.5
C15—C16—H16A	109.5	H36A—C36—H36B	109.5
C15—C16—H16B	109.5	C35—C36—H36C	109.5
H16A—C16—H16B	109.5	H36A—C36—H36C	109.5
C15—C16—H16C	109.5	H36B—C36—H36C	109.5
			
C2—N1—C1—O1	−175.40 (11)	C20—N8—C19—O3	−178.29 (12)
C2—N1—C1—C4	3.58 (18)	C20—N8—C19—C22	2.09 (18)
C3—N2—C2—N3	178.60 (12)	C21—N7—C20—N9	179.71 (12)
C3—N2—C2—N1	−1.22 (18)	C21—N7—C20—N8	−0.80 (19)
C8—N3—C2—N2	6.88 (19)	C26—N9—C20—N7	−176.98 (12)
C6—N3—C2—N2	−176.70 (11)	C24—N9—C20—N7	−3.01 (19)
C8—N3—C2—N1	−173.30 (11)	C26—N9—C20—N8	3.51 (19)
C6—N3—C2—N1	3.13 (19)	C24—N9—C20—N8	177.48 (11)
C1—N1—C2—N2	−1.2 (2)	C19—N8—C20—N7	−1.6 (2)
C1—N1—C2—N3	178.98 (11)	C19—N8—C20—N9	177.87 (12)
C2—N2—C3—C4	1.1 (2)	C20—N7—C21—C22	2.7 (2)
C2—N2—C3—C5	−179.06 (12)	C20—N7—C21—C23	−175.17 (11)
N2—C3—C4—C1	1.5 (2)	N7—C21—C22—C19	−2.2 (2)
C5—C3—C4—C1	−178.38 (12)	C23—C21—C22—C19	175.57 (12)
O1—C1—C4—C3	175.25 (13)	O3—C19—C22—C21	−179.86 (13)
N1—C1—C4—C3	−3.64 (19)	N8—C19—C22—C21	−0.27 (19)
C2—N3—C6—C7	81.37 (16)	C20—N9—C24—C25	98.62 (15)
C8—N3—C6—C7	−102.13 (15)	C26—N9—C24—C25	−87.03 (15)
C2—N3—C8—C9	87.27 (15)	C20—N9—C26—C27	80.44 (16)
C6—N3—C8—C9	−89.38 (14)	C24—N9—C26—C27	−93.72 (14)
C11—N4—C10—O2	−178.31 (12)	C29—N10—C28—O4	−179.85 (12)
C11—N4—C10—C13	1.82 (19)	C29—N10—C28—C31	1.71 (18)
C12—N5—C11—N6	−178.93 (12)	C30—N11—C29—N12	−179.29 (12)
C12—N5—C11—N4	0.77 (19)	C30—N11—C29—N10	0.44 (18)
C15—N6—C11—N5	175.93 (12)	C33—N12—C29—N11	−174.42 (12)
C17—N6—C11—N5	0.78 (19)	C35—N12—C29—N11	−2.13 (19)
C15—N6—C11—N4	−3.8 (2)	C33—N12—C29—N10	5.84 (19)
C17—N6—C11—N4	−178.93 (11)	C35—N12—C29—N10	178.13 (12)
C10—N4—C11—N5	−1.9 (2)	C28—N10—C29—N11	−2.1 (2)
C10—N4—C11—N6	177.82 (12)	C28—N10—C29—N12	177.67 (11)
C11—N5—C12—C13	0.2 (2)	C29—N11—C30—C31	1.41 (19)
C11—N5—C12—C14	178.47 (11)	C29—N11—C30—C32	−177.92 (11)
N5—C12—C13—C10	−0.2 (2)	N11—C30—C31—C28	−1.7 (2)
C14—C12—C13—C10	−178.29 (12)	C32—C30—C31—C28	177.60 (12)
O2—C10—C13—C12	179.31 (14)	O4—C28—C31—C30	−178.23 (13)
N4—C10—C13—C12	−0.83 (19)	N10—C28—C31—C30	0.09 (19)
C11—N6—C15—C16	89.40 (16)	C29—N12—C33—C34	83.51 (15)
C17—N6—C15—C16	−95.34 (14)	C35—N12—C33—C34	−89.09 (15)
C11—N6—C17—C18	89.04 (15)	C29—N12—C35—C36	100.38 (15)
C15—N6—C17—C18	−86.41 (15)	C33—N12—C35—C36	−86.78 (15)

### Hydrogen-bond geometry (Å, °)

**Table:**

*D*—H···*A*	*D*—H	H···*A*	*D*···*A*	*D*—H···*A*
N1—H1A···O1^i^	0.88	2.02	2.8420 (19)	156
N4—H4A···O3	0.88	1.98	2.8407 (18)	164
N8—H8C···O2	0.88	1.99	2.8420 (18)	162
N10—H10A···O4^ii^	0.88	1.98	2.8248 (18)	162
C6—H6A···O1^i^	0.99	2.22	3.116 (2)	150
C15—H15A···O3	0.99	2.35	3.120 (2)	134
C16—H16B···O1	0.98	2.58	3.507 (2)	158
C26—H26A···O2	0.99	2.31	3.130 (2)	139
C33—H33A···O4^ii^	0.99	2.42	3.143 (2)	130

Symmetry codes: (i) −*x*+2, −*y*+1, −*z*+1; (ii) −*x*+1, −*y*, −*z*.
